# Bivalves rapidly repair shells damaged by fatigue and bolster strength

**DOI:** 10.1242/jeb.242681

**Published:** 2021-10-14

**Authors:** R. L. Crane, J. L. Diaz Reyes, M. W. Denny

**Affiliations:** Department of Biology, Stanford University, Stanford, CA 94305, USA

**Keywords:** Cyclic loading, Functional morphology, Mollusk, Biomineralization, Mussel, *Mytilus californianus*

## Abstract

Hard external armors have to defend against a lifetime of threats yet are traditionally understood by their ability to withstand a single attack. Survival of bivalve mollusks thus can depend on the ability to repair shell damage between encounters. We studied the capacity for repair in the intertidal mussel *Mytilus californianus* by compressing live mussels for 15 cycles at ∼79% of their predicted strength (critically fracturing 46% of shells), then allowing the survivors 0, 1, 2 or 4 weeks to repair. Immediately after fatigue loading, mussel shells were 20% weaker than control shells that had not experienced repetitive loading. However, mussels restored full shell strength within 1 week, and after 4 weeks shells that had experienced greater fatiguing forces were stronger than those repetitively loaded at lower forces. Microscopy supported the hypothesis that crack propagation is a mechanism of fatigue-caused weakening. However, the mechanism of repair was only partially explained, as epifluorescence microscopy of calcein staining for shell deposition showed that only half of the mussels that experienced repetitive loading had initiated direct repair via shell growth around fractures. Our findings document repair weeks to months faster than demonstrated in other mollusks. This rapid repair may be important for the mussels’ success contending with predatory and environmental threats in the harsh environment of wave-swept rocky coasts, allowing them to address non-critical but weakening damage and to initiate plastic changes to shell strength. We highlight the significant insight gained by studying biological armors not as static structures but, instead, as dynamic systems that accumulate, repair and respond to damage.

## Abstract (precis)

**Highlighted Article:** Mussels repair shell damage caused by fatigue within one week and further strengthen shells within one month. Bivalve shells are a dynamic armor, responsive to accumulating weakening damage.

## INTRODUCTION

Many mollusks are equipped with a natural armor – a hard shell that defends against a lifetime of physical and environmental threats. Every survived encounter has the potential to leave permanent damage. Therefore, to survive repeated encounters, shells must either be able to withstand or repair accumulated damage before the shell is exposed to a lethal load. The capacity for repair sets many biomineralized tissues apart from manufactured materials but is not widely studied, either in the context of repeated traumas or especially in non-vertebrate systems, which can differ markedly in structure, function and biology from bone. A shell's ability to defend against repeated threats and its capacity for repair are fundamental to its long-term effectiveness.

The study of mechanical fatigue provides the tools to understand how accumulating damage weakens a structure (reviewed in a biological context in Mach et al., 2007). Microscopic cracks in a material cause stress concentrations; the longer the crack, the more severe the stress (process described by Gosline, 2018). As a result, even a subcritical force can cause these initial micro-fractures to extend, further concentrating stresses at crack tips. This positive feedback can progressively weaken a structure. In this way, subcritical forces applied repeatedly or for an extended duration can ultimately break a shell (Boulding and LaBarbera, 1986; Crane and Denny, 2020; Currey and Brear, 1984; LaBarbera and Merz, 1992).

Mollusks build their own shells and can repair many different types of damage. Scars from damage to the shell's edge are common, documenting survived encounters with predators and continuing growth (Blundon and Vermeij, 1983). Many mollusk species also patch drill holes (Meenakshi et al., 1973; Sleight et al., 2015) or thicken internally in response to external wear or damage (O'Neill et al., 2018; Peck et al., 2018). Although an initial repair response has been documented within hours or days (Mount et al., 2004; Reed-Miller, 1983), studies focusing on mechanical properties of gastropods have not found full repair for weeks to months (LaBarbera and Merz, 1992; O'Neill et al., 2018). Notably, most experimental studies of shell repair focus on fractures or holes that fully pierce the shell, in contrast to the accumulating fracture hypothesized to be associated with repetitive loading.

California mussels (*Mytilus californianus*) are an opportune system in which to study fatigue and repair. Their general biology is well-studied owing to their economic and ecological significance, and they have a simple, tractable morphology – a single shell consisting of two domed valves. The shells possess an external proteinaceous layer, the periostracum, covering layers of crystals of calcium carbonate in organic matrix: an outer prismatic layer, a middle layer of bricklike nacre and a thin, innermost prismatic layer (Dodd, 1964; Taylor et al., 1969). Mussel shells experience fatigue on potentially biologically relevant timescales (Crane and Denny, 2020), although repair in response to fatigue has not been directly studied.

In this study, we asked: are California mussels capable of reversing the loss of strength from fatigue loading? If so, how long does this repair take? And finally, is there physical evidence of accumulating damage to and repair of shells? To answer these questions, we repetitively loaded live mussels (henceforth ‘stressed’ mussels), and 0, 1, 2 or 4 weeks later, we measured and compared the strengths of stressed mussel shells with the strengths of mussel shells that had not experienced repeated experimental loading. We found that fatigue loading weakened shells, but that mussels were able to repair within a single week. Further, we found that the shells of mussels loaded at higher forces for their size were stronger after four weeks than the shells of mussels loaded at lower forces. We identified patterns of fracture and repair using several methods of microscopy. We found increased fracture in stressed shells; however, only in a subset of these shells could we identify a direct repair response around fractures.

## MATERIALS AND METHODS

### Experimental design

Whole live mussels (*Mytilus californianus* Conrad 1837) were repetitively loaded in compression (‘stressed’ mussels) then housed in a flow-through seawater system. At each of four subsequent time intervals (0, 1, 2 and 4 weeks), a subset of mussels was removed and killed. Their valves were either strength tested, to quantify changes in shell mechanical properties, or their valves were prepared and inspected using multiple kinds of microscopy to visualize evidence of damage and repair (Figs S1 and S2). As a control, live mussels were collected, maintained with the stressed mussels, and analyzed in the same ways but were not exposed the repetitive loading treatment (henceforth ‘non-stressed’ mussels). Controlling for size, mussels were randomly assigned a treatment (stressed or non-stressed), a final test group (strength-testing or microscopy) and a test week (0, 1, 2 or 4 weeks).

### Animal collection and maintenance

Mussels (*N*=536; length: 34.2±4.6 mm, mean±s.d.; range: 23.7–44.5 mm) were collected at Hopkins Marine Station, Pacific Grove, CA, USA (Scientific Collecting Permit no. S-190720016-19072-001), from a single site (0.3–0.9 m above MLLW, 36.62199°N, 121.90536°W). We included only mussels with minimal damage, defined as a majority of the periostracum intact and no external damage that extended deep enough to make the nacreous layer visible.

Mussels were housed in a flow-through seawater system under shaded, natural lighting. Tanks filled and drained on an approximate 8.25 h high:4.17 h low cycle, resulting in a 50.4 min daily shift. The period of exposure matched the average daily exposure across the duration of the experiment for the average tidal height from which the mussels were collected. Once daily at high tide, mussels were immersed for 1 h in a dilution of marine microalgae concentrate (Shellfish Diet 1800, Instant Algae, Reed Mariculture, CA, USA).

### Initial morphological measurements

At the start of the study, we measured the length, width and height of each live mussel (Crane and Denny, 2020). The animals were then patted dry and weighed. Mussels were identified with a small waterproof tag (2.5 mm diameter circle), which was adhered to the dorsal posterior quadrant of the right valve with a dot of superglue.

### Fatigue treatment

**Fig. 1.**
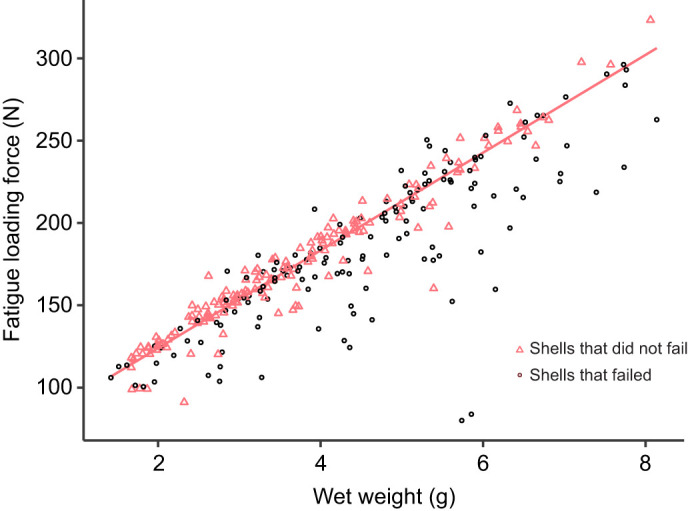
**Fatigue loading protocol for whole live interidal mussels** (*Mytilus californianus*). Mussels were compressed for 15 cycles to a force that depended on their initial wet weight. The shells of 141 out of 305 mussels failed catastrophically during fatigue loading (black circles). For mussels whose shells did not break (pink triangles), the resulting relationship between the fatigue loading force, defined as the median maximum force for all cycles, and the wet weight is defined by the linear regression equation: applied force (*N*)=64.6+29.7×wet weight (g) (*F*_1,162_=2284, *P*<0.0001, *R*^2^=0.93).

Whole live mussels were repeatedly loaded in compression one at a time in a materials testing machine for 15 cycles to a predetermined force that depended on initial mussel wet weight (Fig. 1; force range: 80–323 N). Mussels were visually inspected by eye after fatigue loading, and any valve that was shattered or otherwise visibly fractured externally was considered to have experienced catastrophic failure. Any mussel for which at least one valve experienced catastrophic failure was excluded from further testing. For mussels whose shells did not break during fatigue loading, the applied force can be described according to the linear regression equation: applied force (N)=64.6+29.7×wet weight (g) (*F*_1,162_=2284, *P*<0.0001, *R^2^*=0.93). The range of forces was determined based on previous work to cause critical damage to approximately half of shells (Crane and Denny, 2020) and the resulting range resembled the range of crushing forces from many shell-crushing crabs in the Pacific Northwest, USA (Taylor, 2000), which the California mussel could encounter.

Mussels were compressed for 15 cycles between two flat plastic plates. The materials testing device applied a compressive force from above with a hydraulic ram, with the mussel resting on a force plate with its aperture parallel to both plates (details about force sensor and materials testing device in Crane and Denny, 2020). The valve (left or right) in contact with the force plate was randomly determined. Mussels were held in place with a small piece of flexible modeling clay. Shells were loaded and unloaded to a position at which the compressed force plate provided the target force, and the duration of each loading and unloading cycle was consistent across all cycles for a given mussel and ranged across mussels from 0.31 to 0.67 s. The applied force for an experiment is defined as the median of all cycle force maxima, excluding the cycle during which the valve broke (unless the valve broke during the first cycle). For mussels that survived at least three cycles, the standard deviation of the maximum force for each cycle (excluding the cycle during which the mussel shell failed catastrophically, if it failed) averaged 8 N (range: 1–30 N) with 90% of trials having standard deviations below 16 N. Dividing the standard deviation for each mussel by the applied force as defined above, the average was 4.6% (range: 0.8–22%) with 90% of trials below 9%.

### Final morphological measurements

Subsequent to fatigue loading and the recovery period, but before strength testing or microscopy, mussels were dissected by inserting a scalpel into the thin gap at the ventral side of the shell, severing the posterior adductor muscle, and gently opening the valves. The length, height and width of each valve were measured. Acting gently and with care so as not to damage the shell, the soft tissue was pushed out of the shell with a round and blunt nickel spatula. The valves were patted dry and weighed, and the tissue was dried at 60°C until the mass stabilized. Valves to be strength-tested were stored immersed in saltwater and tested the same day. Valves used in microscopy were stored in Falcon Tubes, wrapped in aluminium foil (to limit light exposure), placed in a refrigerator, and imaged within 2 days.

### Strength testing

Strength testing was performed using the same materials testing device as the initial fatigue treatment. The left and right valves of each mussel were tested separately. From each loading curve, the one-time breaking force was identified as the force at catastrophic failure, which we refer to as ‘strength’. Traditionally, ‘strength’ refers to the stress at failure, a force per area. However, quantifying stress distributions across the mussel shell and relevant cross-sectional areas was beyond the scope of this study.

### Microscopy

A group of mussel shells were imaged with two kinds of microscopy. First, the internal surface was inspected with a light microscope (ZEISS V12 Discovery Stereoscope, Oberkochen, Germany; with a ZEISS Achromat S 1.0× objective, FWD 69 mm; photographs through a 1.6× SLR tube) for evidence of fracture and repair. Second, calcein (Sigma-Aldrich, St Louis, MO, USA), a fluorescent dye that binds to calcium, was used to distinguish areas of shell growth under epifluorescence with a GFP filter (X-Cite Series 120, EXFO, Quebec City, Canada). By maintaining live mussels in a calcein bath, the dye bound to new shell as it was deposited, distinguishing shell deposition that occurred while the mussel was in the bath (Le Moullac et al., 2018; van der Geest et al., 2011).

Accounting for shell size, treatment and recovery period, mussels were randomly assigned to one of three staining regimes: an unstained regime; a stained after dead regime; and a stained while live experimental treatment (Fig. S2). The unstained group remained in seawater without calcein until killed and imaged, acting as a control for the extent of fluorescence in the absence of any calcein (*N*=2 non-stressed mussels week 0; *N*=1 stressed mussel subsequent weeks). The mussels stained after death were killed 1 week before imaging. The valves were separated, and the internal tissue was removed. The shells were then immersed for 1 week in the calcein bath before being imaged. These mussels acted as no-growth controls, indicating the quantity and distribution of fluorescence on shells that were not actively growing (*N*=5 non-stressed week 0; *N*=4 stressed subsequent weeks). Mussels in the stained-while-alive group were moved live to the calcein bath 1 week before being killed and imaged, allowing direct comparison of growth patterns for stressed and non-stressed shells (*N*=5 non-stressed week 0; *N*=8 stressed and 6 non-stressed subsequent weeks).

The calcein bath was a 41.5 liter recirculating system. It was filled with seawater from the flow-through system and calcein (0.01 g l^−1^). Water rose and fell as with the flow-through system. Water level and salinity were maintained by adding deionized water once per day. Tanks were stored in the laboratory (temperature range: 19–25°C). Mussels were fed 0.5 ml of algal suspension three times per week. The calcein tank was emptied and refilled for every new set of mussels.

### Statistical analyses

Statistical analyses were conducted in R (version 3.3.2, https://www.r-project.org/). Data and code are available on Mendeley Data with doi:10.17632/hphn2m6r27.1.

#### Timeline of mussel shell repair after fatigue

We assessed fatigue and repair by comparing shell strength of stressed and non-stressed mussel shells. For every mussel, we measured the strength of both valves, then considered only the strength of the weaker valve as representative of overall shell strength. We could not directly compare the strength of the stressed and non-stressed groups because during fatigue loading, 46% of the compressed mussels (141/305 mussels) were excluded owing to catastrophic failure of at least one valve. Thus, the weakest shells were removed from further consideration. In contrast, the non-stressed group contained all initially collected mussels. To account for this discrepancy, after measuring the strength of the non-stressed shells, we subsampled by removing the weakest 46% of shells, accounting for size. Specifically, for the non-stressed shells at each recovery duration, we fitted a multiple regression of shell strength in terms of final shell mass. We excluded the 46% of mussels with the most negative residuals (*N*=91 mussels) and used the remaining 54% (*N*=109 mussels) as the control comparison (henceforth ‘control’ shells).

We compared the strengths of control and stressed shells at each of the four recovery durations (0, 1, 2 and 4 weeks). For each time point, we conducted an analysis of covariance (ANCOVA) of the shell's final strength in terms of treatment (stressed or control) with covariates of the shell's final mass and domedness (ratio of width to length) (Crane and Denny, 2020). Before conducting the ANCOVA, we tested for homogeneity of slopes, testing the interactions between treatment and mass as well as between treatment and domedness. For shells tested 1 week after fatigue loading, a significant interaction emerged between domedness and treatment (*P*<0.05). However, this interaction introduced significant multicollinearity, with a tolerance of only 0.0035 (a measure ranging from 0 to 1 describing how much new information was provided by introducing the interaction). Owing to the lack of substantial information added by including this term, we moved forward with the ANCOVA for the first week, excluding the interaction term. No other interactions were significant (*P>*0.05).

We used two-way ANOVAs to test whether test week or treatment varied with either initial shell length or initial mussel wet weight. Because 46% of stressed mussels broke, we assessed the size distributions for both the initial and the reduced groups after accounting for mussel breakage. Additionally, we assessed which shells broke during fatigue treatment by fitting a binomial generalized linear model of whether a shell broke in terms of initial mussel wet weight, the shell's domedness, and the fatigue force residual – the deviation in the applied force from the typical fatiguing force for a mussel of that size (i.e. the residual of the regression of fatigue force in terms of mussel wet weight; Fig. 1).

#### Benefits of experiencing fatigue

We tested whether mussels that had experienced relatively larger fatiguing forces differed in final strength. Examining only stressed shells and separately for each recovery duration, we fit multiple regression models of final shell strength in terms of final shell mass and domedness and the fatigue force residual – the deviation in the fatiguing force from the typical fatiguing force for a mussel of that size (i.e. the residual of the regression of fatigue force in terms of mussel wet weight; Fig. 1). We reduced the model, removing non-significant predictors. We also ran an initial statistical model that included interaction terms of each morphological variable with the fatigue force residual and excluded these terms because they were not significant (*P*>0.05).

#### Cost of repairing fatigue damage

We used mussel soft tissue mass to quantify the cost of repair. First, we tested for an effect of being maintained in the lab. For all non-stressed mussels, we conducted an ANOVA on the ratio of soft tissue to shell mass in terms of time in the lab, followed by *post hoc* Tukey contrasts between all pairs of testing weeks. Owing to deviations from normality for the second week, we repeated these analyses with non-parametric tests: a Kruskal–Wallis test followed by pairwise comparisons with Wilcoxon rank sum tests with a Bonferroni correction.

Second, we assessed whether the amount of soft tissue differed between stressed and control mussels by, separately for each week, running a Welch two-sample *t*-test comparing the ratio of soft tissue to shell mass between stressed and control mussels. Owing to deviations from normality for the second and fourth weeks, we repeated all analyses with non-parametric Wilcoxon rank sum tests.

#### Microscopy: internal evidence of damage and repair

We tested whether fractures were more common in stressed shells than non-stressed shells with a chi*-*squared test. The internal surfaces of many shells had patches that had clearly defined borders and differed in color from the surrounding shell; we identified these patches and compared their prevalence in stressed and non-stressed shells with a chi-squared test.

## RESULTS

### Timeline of mussel shell repair after fatigue

**Fig. 2.**
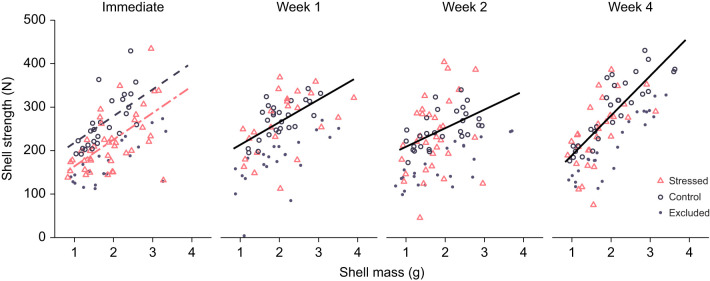
**Mussel shells were weakened by fatigue loading but repaired within 1 week.** Immediately after treatment, stressed shells (pink triangles, dotted/dashed regression) were significantly weaker than control shells (hollow gray circles, dashed regression) after accounting for shell mass and morphology (Table 1). After 1, 2 and 4 weeks, they did not differ significantly (Table 1), and therefore only a single regression line is plotted (solid black). Control shells include only a subset of all non-stressed shells, to account for the 46% of stressed mussels that broke during treatment. The excluded non-stressed shells are plotted (small gray circles). Regressions shown with domedness held constant at average value. Stressed: *N*=40 mussels immediate, *N*=24 week 1, *N*=32 week 2, *N*=29 week 4; control: *N*=26 immediate, *N*=26 week 1, *N*=28 week 2, *N*=29 week 4; excluded non-stressed: *N*=22 immediate, *N*=22 week 1, *N*=23 week 2, *N*=24 week 4.

Immediately after fatigue loading, mussel shells were significantly weaker than non-stressed control shells by an average of 54 N (Fig. 2, Table 1). However, the shells of stressed mussels that were allowed 1, 2 or 4 weeks to recover did not differ significantly in strength from control shells (Fig. 2, Table 1). After all recovery durations, shells that were more massive or more domed (greater ratio of width to length) were significantly stronger except after 4 weeks, when domedness was not significant (Table 1).

**Table 1.**
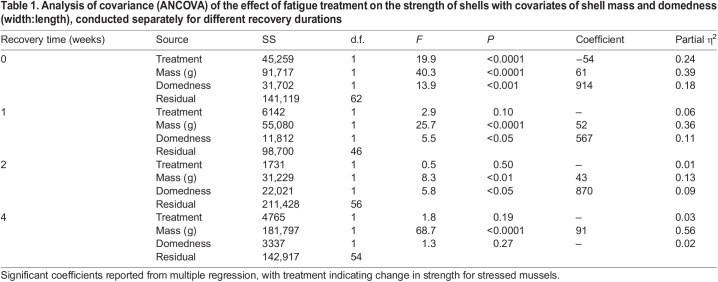
Analysis of covariance (ANCOVA) of the effect of fatigue treatment on the strength of shells with covariates of shell mass and domedness (width:length), conducted separately for different recovery durations

Neither mussel length nor wet weight differed between stressed and non-stressed groups and across test-weeks, as initially assigned (Table S1). After accounting for mussels whose shells broke during fatigue treatment, control mussels were about 3% longer initially than stressed mussels with no effect of test week (Table S1). Initial mussel wet weight was still not associated with either treatment or test week (Table S1).

Shells with a greater initial wet weight were more likely to break during fatigue loading (binomial generalized regression: *P*<0.001, β=0.32, s.e.=0.09, odds ratio=1.38, *N*=304 mussels), and shells that were more domed or were stressed at relatively higher forces were less likely to break during fatigue loading (domedness: *P*<0.01, β=−16.98, s.e.=6.06, odds ratio=4.22×10^−8^; fatigue force residual: *P*<0.0001, β=−0.045, s.e.=0.009, odds ratio=0.956).

### Benefits of experiencing fatigue

**Fig. 3.**
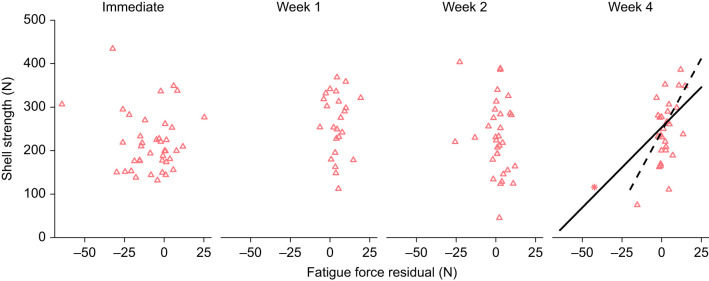
**Within 4 weeks, mussels that experienced fatigue loading at greater forces for their size were stronger.** The fatigue force residual (*x*-axis) was calculated from the regression modeling the magnitude of the fatigue force in terms of mussel wet weight (Fig. 1), and thus reports the magnitude of difference between the actual loading force and the expected force based on mussel size. For example, a residual of −20 N would indicate the mussel was loaded to 20 N less than the average loading force for a mussel of its size. 0, 1 or 2 weeks after fatigue loading, the magnitude of the residual was not associated with shell strength, but after 4 weeks, mussels with larger fatigue force residuals (i.e. that initially experienced larger fatiguing forces for their size) were stronger (Table 2). Asterisk in week 4 indicates an outlier with high influence (Cook's distance>1). Black lines indicate multiple regression models with mass held constant at the average; solid line represents the model including the high influence shell, and dashed line indicates the model without it. *N*=40 mussels immediate, *N*=24 week 1, *N*=32 week 2, *N*=29 week 4.

Up to 2 weeks after fatigue treatment, the fatigue force residual (the difference between the observed and expected fatigue force accounting for mussel size) was not associated with the shell's strength (Fig. 3, Table 2). After 4 weeks, however, shells that had experienced greater fatiguing forces for their size were stronger than shells that had been stressed at lower forces (Fig. 3, Table 2). The effect of shell morphology on strength varied, with larger shells being stronger after 0, 1 and 4 weeks, and more domed shells being stronger after 0, 1 and 2 weeks (Table 2). One mussel from the 4-week recovery group was repetitively loaded at a particularly low force (Fig. 3) and had high influence on the final model (Cook's distance=3.9). Excluding this shell altered coefficients but not conclusions of the final model (Fig. 3, Table 2), and no remaining points had high influence (Cook's distance<1).

**Table 2.**
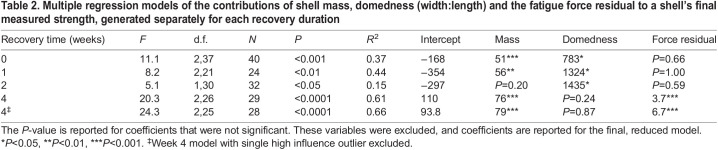
Multiple regression models of the contributions of shell mass, domedness (width:length) and the fatigue force residual to a shell's final measured strength, generated separately for each recovery duration

### Cost of repairing fatigue damage

**Fig. 4.**
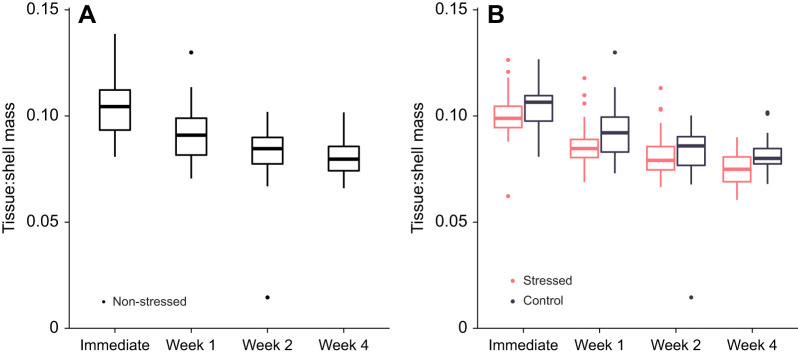
**Dry internal tissue mass relative to shell mass.** (A) Indicating some cost of being stored in the lab, across 4 weeks, the relative mass of dry internal tissue decreased for non-stressed mussels (ANOVA: *F*_3,213_=47, *P*<0.0001, *N*=47 mussels immediate, *N*=54 week 1, *N*=57 week 2, *N*=59 week 4). *Post hoc* Tukey contrasts revealed significant differences between all combinations of weeks (*P*<0.001), except between the second and fourth weeks, which were not significantly different (*P*=0.67). (B) Comparing stressed mussels (pink) with control mussels (gray) separately at each point, stressed mussels had slightly but significantly less soft tissue 1 (*t*=−2.4, d.f.=49.5, *P*<0.05, *N*=63) and 4 weeks after fatigue loading (*t*=−3.4, d.f.=57.4, *P*<0.01, *N*=71), but differences were not significant immediately (*t*=−1.0, d.f.=52.0, *P*=0.32, *N*=65 mussels) or 2 weeks after fatigue loading (*t*=−0.46, d.f.=39.4, *P*=0.64, *N*=73).

The evaluation of the cost of being stored in the lab indicated that across 4 weeks in lab, the ratio of soft tissue to shell mass of non-stressed mussels decreased (ANOVA: *F*_3,213_=47, *P*<0.0001, *N*=217 mussels; non-parametric: Kruskal–Wallis χ^2^=88.9, d.f.=3, *P*<0.0001; Fig. 4A). *Post hoc* Tukey contrasts revealed significant differences between all pairwise combinations of weeks (*P*<0.001), except between the second and fourth weeks, which were not significantly different (*P*=0.67); for all significant between-week comparisons, the later week had relatively less soft tissue. The non-parametric *post hoc* Wilcoxon tests found the same significance patterns.

Analyses of whether stressed mussels suffered a greater loss of tissue than control mussels found that, although the ratio of soft tissue to shell mass did not differ between control and stressed mussels immediately (Welch two-sample *t-*test: *t*=−1.0, d.f.=52.0, *P*=0.32, *N*=65 mussels) or 2 weeks after fatigue loading (*t*=−0.46, d.f.=39.4, *P*=0.64, *N*=73), stressed mussels had relatively less soft tissue 1 week (*t*=−2.4, d.f.=49.5, *P*<0.05, *N*=63) and 4 weeks after fatigue loading (*t*=−3.4, d.f.=57.4, *P*<0.01, *N*=71). These differences at 1 and 4 weeks were significant, though small; stressed mussels had, on average a tissue:shell mass ratio 0.007 and 0.006 less, respectively, than control mussels. This corresponds to the soft tissue of an average stressed mussel weighing ∼0.01 g, or 7%, less. Non-parametric Wilcoxon rank sum tests produced the same significance patterns (immediate, *W*=400, *P*=0.15; week 1, *W*=307, *P*<0.05; week 2, *W*=479, *P*=0.09; week 4, *W*=351, *P*<0.01).

### Microscopy: internal evidence of damage and repair

**Fig. 5.**
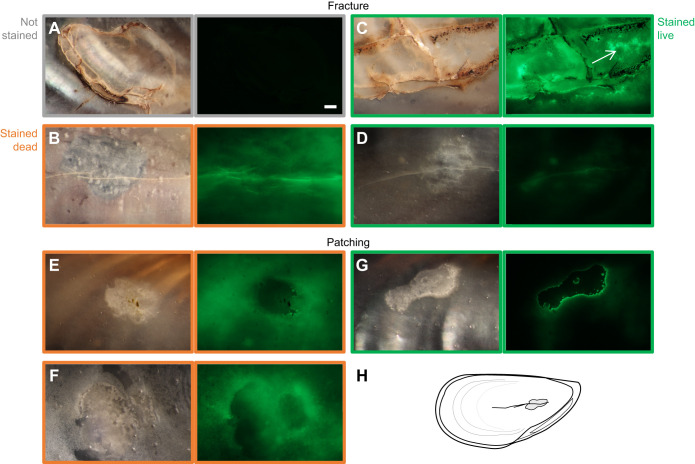
**Fracture and repair of the central internal surface of stressed mussels.** Light (left) and epifluorescent (right) micrographs are of the same location, and all fluorescent images were captured at the same settings. Border colors indicate staining regime: unstained in gray (A); stained dead in orange (B,E,F); stained live in green (C,D,G). (A,B,C,D) Fractures of variable morphologies were common in stressed shells and sometimes associated with areas of brown growth. (A) Unstained shells did not autofluoresce, including across extensive fracture and associated brown growth. (B–D) In stained shells, fractures generally fluoresced brightly. (B) In shells stained dead, these fractures were usually surrounded by a diffuse fluorescence across the internal shell surface. (C,D) In contrast, shells stained live showed either: localized increased fluorescence distant from the fracture (C, arrow) or minimal response (D). (E–G) Patches of thickened shell with defined borders were common (E,F) with diffuse fluorescence in mussels stained dead, and (G) a consistent pattern of a dark patch with a defined border for mussels stained live. (H) Schematic illustrating shell orientation (anterior–posterior axis consistent, though images include both left and right valves) and a representative patch and fracture. Scale bar: 1 mm, applicable to all microscopy images.

First, we considered the overall extent of fluorescence. Except for the hinge, the inner surface of the unstained shells did not autofluoresce (Fig. 5A). Valves stained after the mussel was killed generally fluoresced brighter across the whole internal surface (Fig. 5B,E,F) than mussels that were stained while alive (Fig. 5C,D,G). This increased fluorescence likely occurred because dead shells were exposed directly to the calcein bath, whereas the inner shell surface of live mussels was protected by the mantle.

Fractures were often visible under visible-light microscopy and were significantly more common in stressed shells than in non-stressed shells (χ^2^=10.6, d.f.=1, *P*<0.01; 14/39 stressed, 1/30 non-stressed shells; Fig. 5A–D). The extent and shape of fractures varied from single short, mostly linear cracks, to webs of damage spreading across the internal surface. Fractures on stressed mussel shells were found most commonly across the central dome of the shell (*N*=13 valves), sometimes on the posterior edge (*N*=4 valves) and, on one occasion, on the ventral edge spreading toward the center of the dome. The one damaged non-stressed valve was fractured on the posterior edge.

We examined fractures and surrounding shell for evidence of repair. Fractures fluoresced in all stained mussels (Fig. 5B–D). However, fluorescence in the area surrounding the fracture differed depending on whether the mussel was stained alive or dead. Of mussels stained alive, increased fluorescence around the fracture at ∼1 mm distance, not associated with other features, was present in 5 of 11 fractured, stressed valves (present: Fig. 5C; absent: Fig. 5D). The one fractured but non-stressed mussel also showed increased fluorescence around the fracture (similar to Fig. 5C). In contrast, among mussels that were stained dead, the area around the fracture did not show a similar localized increase in fluorescence relative to background levels (Fig. 5B). Additionally, brown growth had spread over or around cracks in 8 of 18 stressed valves with fracture and the one non-stressed valve with fracture (Fig. 5A,C). These small brown regions resembled periostracum in color, were always directly associated with fractures and notably, did not fluoresce, suggesting they are not primarily calcium carbonate. There is no suggestion that the number of shells showing a direct response to fracture varied across the 4 weeks (increased fluorescence around fracture in mussels stained live: 3/4 valves at week 1, 3/6 week 2, 0/2 week 4; brown growth in fractured mussels: 3/7 valves at week 1, 5/8 week 2, 1/4 week 4).

Not associated with presence of fractures on the shell, we frequently found areas of thickened shell deposition on the internal surface of the shell, which we refer to as patches. These patches had clearly defined borders, were consistently identifiable by a distinctive matte quality that differed from the shine and opalescence of the usual internal surface of mussel shells, and ranged in color from off-white to gray to navy (Fig. 5E–G). They were likely initiated before our experiment. The frequency of these patches did not differ between stressed and non-stressed shells (χ^2^=0.09, d.f.=1, *P*=0.76; 30/39 stressed shells, 24/30 non-stressed shells; Fig. 5E–G). Additionally, the patches showed a distinctive staining pattern. Generally, in mussels stained live, the patch itself was darker than the surrounding shell, and its edges were clearly defined and brightly fluorescent (Fig. 5G). This fluorescence pattern was seen extensively in stressed (33/35 valves) and non-stressed (30/34 valves) mussels with patches. In contrast, of mussels stained dead, only 1 of 20 valves with patches matched this fluorescence pattern. Instead, many patches of mussels stained dead fluoresced more than the surrounding shell (*N*=6 valves), or had edges that were either unstained or poorly defined (*N*=18 valves, Fig. 5E,F). The differences between mussels stained live and those stained dead suggest that the fluorescence pattern in the stained live group indicates new shell deposition at the patch edge.

## DISCUSSION

Mussels that survived a significant, fatiguing load were initially weakened but repaired within 1 week, such that they were as strong as non-stressed control mussels (Fig. 2, Table 1). Within 1 month, mussels that had experienced a relatively high fatiguing force had bolstered their strength relative to mussels that had experienced fatigue loading at a lower force, suggesting that sufficient fatigue loading can trigger a compensatory response resulting in an ultimately stronger shell (Fig. 3, Table 2). The presence of fractures on the internal surface of stressed shells supports the hypothesis that accumulating damage is a mechanism of fatigue-caused weakening; however, as a mechanism of repair, we only sometimes saw evidence of growth directly around these fractures. Finally, we found that stressed mussels had 7% less soft tissue than non-stressed mussels 1 and 4 weeks after fatigue loading, providing some preliminary evidence of a cost to repair.

**Fig. 6.**
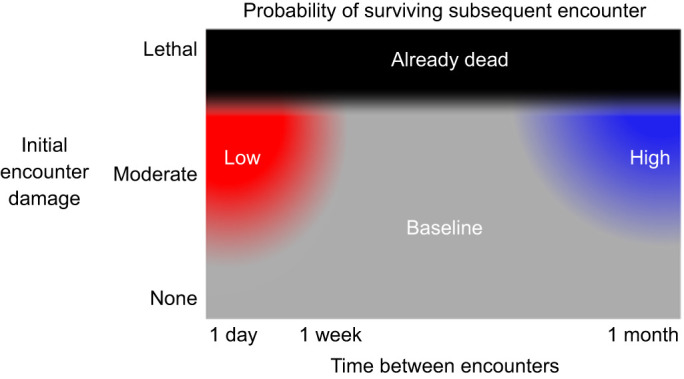
**Although initially weakened by mechanical fatigue, mussels’ probability of surviving their next encounter rapidly improves.** Within 1 week, stressed mussels are as strong as non-stressed mussels and within 1 month, they have increased their defenses against future attacks.

Although mussels are susceptible to fatigue loading from threats like predators and wave-hurled debris (Crane and Denny, 2020; Shanks and Wright, 1986), their remarkably fast repair and compensatory responses – on average less than a week and a month, respectively – narrow the ecological contexts in which fatigue becomes relevant. As has been demonstrated previously, predators that apply repeated forces in a single encounter, like crabs, may rely on fatigue to fracture a mussel shell (Boulding and LaBarbera, 1986). However, within 1 week, a mussel that survives a predation attempt would not be at increased risk in the next encounter, and it could in fact be at decreased risk in an encounter 1 month later (Fig. 6). These findings are based on analyses of changes to average shell strength. Fatigue loading and repair could also affect variance in shell strength; more specifically, does experiencing repeated loading increase the variance in shell strength across mussels, which then decreases again as the more damaged shells are repaired? Unfortunately, these data are not well suited to tests of variance, because of the large proportion of shells that broke during fatigue loading. Whether due to universal changes in strength or more idiosyncratic changes between mussels resulting in changes to variance that shift the average, the rapid repair response that we documented could provide a significant advantage against threats like predators and the hydrodynamic forces imposed by large storms that occur at hard-to-predict intervals.

The timeline of repair in the California mussel exceeds expectations. Other hard-shelled mollusks can take weeks to months to repair fatigue damage (LaBarbera and Merz, 1992; O'Neill et al., 2018). In response to perceived threats, many mollusks will alter shell form by, for example, thickening or strengthening the shell as a whole or in localized regions such as the aperture of snail shells; these alterations can take 2–3 months and have primarily been examined in response to environmental or chemical cues (Appleton and Palmer, 1988; Bourdeau, 2010; Bourdeau and Padilla, 2019; Currey and Hughes, 1982; Leonard et al., 1999). In contrast, we show a direct mechanical response to experienced damage. The magnitude of response with a longer timeframe and ongoing mechanical damage is yet to be determined, as is the mechanism by which mussels identify damage and trigger a response.

Although mussels repaired quickly, we found evidence that this repair came at a cost. Mussels stored in the lab showed a loss of soft tissue (Fig. 4A), and stressed mussels further had ∼7% less soft tissue than control mussels 1 and 4 weeks after fatigue loading (Fig. 4B). Whether due to a loss of soft tissue or an increase in shell deposition, this difference suggests a potential cost of repair in terms of overall health and reproductive capacity, possibly because of the cost of producing shell (Palmer, 1992). The strength of this finding is limited as this effect was not significant 2 weeks after fatigue loading. We cannot explain why we found differences between stressed and control mussels on some but not all weeks. We note, however, that the significant differences in soft tissue were found at times corresponding to mechanical changes: initial repair within 1 week and compensatory strengthening after 4 weeks. Despite costs in soft tissue, microscopy revealed that stressed and non-stressed mussels both continued to deposit shell in similar patterns around existing patches, highlighting that even a significant fatiguing force did not fully interrupt normal shell growth patterns. Further work is required to parse the variability in the effect on soft tissue and the long-term repercussion of repairing fatigue damage.

Although strength testing indicated shells had repaired, calcein staining and microscopy revealed potential repair in only some of the stressed shells. Brown growth spread near and across fractures in almost half of damaged shells (9/19 valves) and inferred shell deposition increased adjacent to fractures in half (6/12 valves), suggesting that improved strength in stressed shells could be due to thickening and direct repair in some but not all mussels. Determining the extent to which the inferred shell deposition would expand and cover fractures will require longer-term monitoring. As for the specific nature of the brown growth, identifying it would require chemical analysis and more advanced microscopy than was performed in this study. It is possible this growth is or is similar to periostracum; Meenakshi and colleagues (1973) demonstrated that the mussel *Mytilus edulis* repairs holes drilled in its shell by first covering the hole with periostracum before initiating shell deposition, and they also documented one instance of periostracum inside the nacreous layer of a valve that was not experimentally damaged. Research in other mollusks has documented repair of holes beginning instead with formation of a non-periostracum collagenous (Su et al., 2004) or brownish chitinous layer (Cho and Jeong, 2011). Although repair visible by microscopy could not fully explain the mechanical results, repairing limpets show a similar mismatch between timelines of mechanical evidence of repair and extent of internal thickening (O'Neill et al., 2018). In the case of our mussels, other methods of visualizing repair might reveal patterns of direct response not visible with calcein staining. Mussels may have a self-healing or compensatory mechanism that does not involve shell deposition. For example, nacre has remarkable strain-hardening and crack-arresting properties (Barthelat and Espinosa, 2007; Espinosa et al., 2011); although it is unclear how these microstructural effects would scale to the entire shell.

Shell morphology and fracture patterns can provide insight into which shells fail and when. In accord with previous work (Crane and Denny, 2020), we found shells that were larger (more massive) and/or more domed to usually be stronger and more fatigue resistant (Tables 1, 2). One test produced the opposite trend – larger shells were more likely to break during fatigue treatment – which we attribute to our overcompensating for shell size when determining the target fatiguing force. Most fractures spread across the internal surface of the shell dome near its apex (13/19) or across the posterior edge (5/19). Fractures across the dome varied in size and shape, including relatively linear fractures running along the anterior–posterior axis (Fig. 5B,D), fractures branching from the apex, and webs of fracture (Fig. 5A,E). For a shell under compression at the apex, the inner surface of the apex will experience tension, and mussel shell, although very effective in compression, tends to fail in tension (Currey, 1989). Prevalent damage at the posterior shell edge may be due to both its thinness and the often uneven aperture surface, which could concentrate stresses and lead to fracture. This weakness aligns with predator behavior, as predators often target the posterior edge, especially when the mussel is too large to crush outright (Elner, 1978). The posterior edge was also notably the only site of fracture damage in the non-stressed shells, and therefore the only damage definitively attributable to experiences in the field that preceded the experiment.

We have shown that substantial fatigue loading, as from a failed predator attack, can weaken a mussel shell through accumulating fracture. However, mussels are, on average, able to repair to initial strength within a single week. Furthermore, having experienced greater fatigue loading spurs mussels to bolster shell strength. This repair may come at a cost, resulting in less soft tissue for a mussel's size. The mechanisms underlying the improvements in strength are still unknown as some, but not all, fractured shells exhibited a visible, direct response. The timelines of fatigue and repair are fundamental to ecology, as bivalves rely on the same armor to contend with repeated threats throughout their lives. The capacity for repair is of further interest in biomineralized tissues, as it distinguishes many of them from manufactured materials. Studies of fatigue and repair in bone have made significant contributions to medicine and biomaterials (Arola et al., 2010; Taylor, 2018). However, non-vertebrate systems remain understudied. Our work shows an impressive and rapid capacity for repair in one bivalve species, and it highlights the necessity of considering many armors as dynamic systems, repairing and responding to repeated threats.

## Supplementary Material

Supplementary information
