# Supplementary material for: Bivalves rapidly repair shells damaged by fatigue and bolster strength
Source: J Exp Biol. 2021 Oct 14;224(19):jeb242681. doi: 10.1242/jeb.242681 (PMC8541735; doi:10.1242/jeb.242681)
Supplement: Supplementary information [file jexbio-224-242681-s1.pdf]

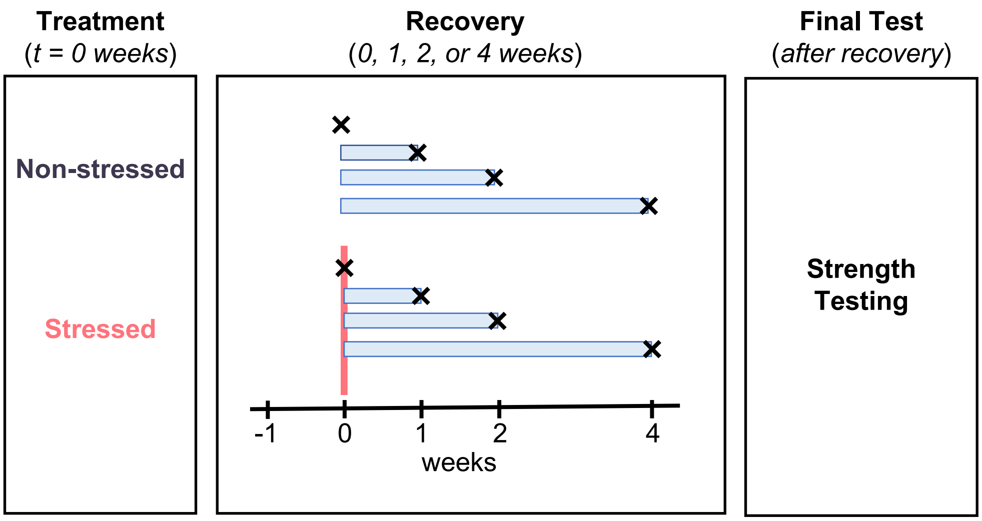

Fig. S1. Experimental design for mussels that were strength tested. Mussels were divided into two treatment groups (stressed or non-stressed), and the treatment was administered (pink line) at the start of the experiment (*t*=0 weeks). Mussels were then stored in a flow-through aquarium system (blue rectangle) for an assigned recovery period (0, 1, 2, or 4 weeks). At the end of their recovery period, mussels were killed (black X), and their valves were strength tested that day.

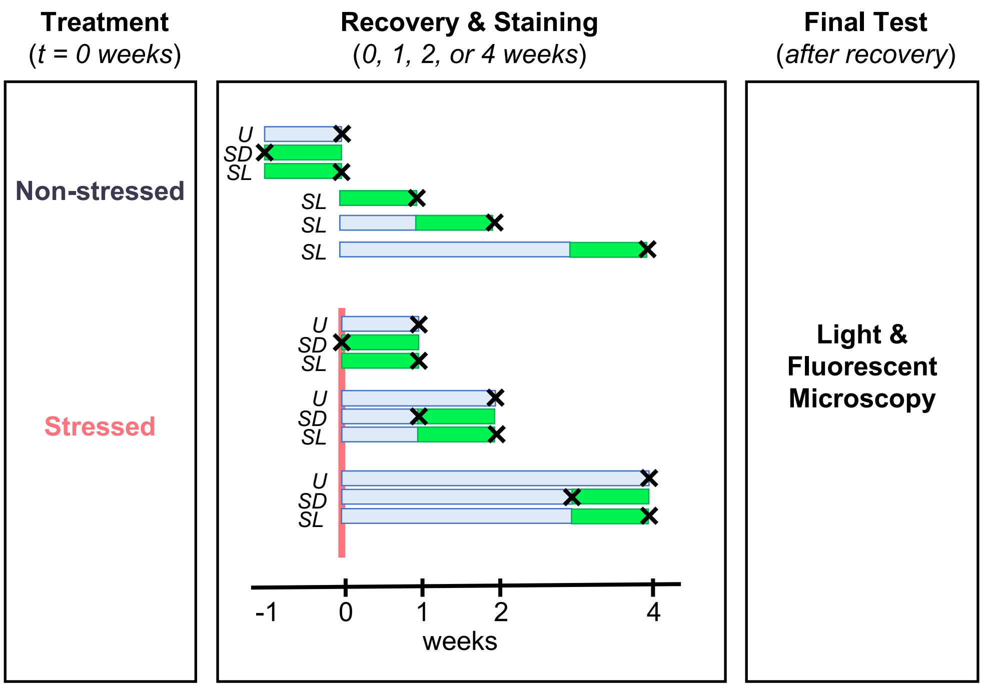

Fig. S2. Experimental design for mussels inspected with microscopy. Mussels were divided into two treatment groups (stressed or non-stressed) and assigned a recovery duration (0, 1, 2, or 4 weeks). The treatment was administered (pink line) at the start of the experiment (*t*=0 weeks). Mussels were also assigned one of three staining regimes. (1) In the unstained regime (U), mussels were stored in a flow-through aquarium system (blue rectangle) throughout their assigned recovery period after which they were killed (black X) and imaged. (2) In the stained while dead (SD) regime, mussels were maintained in a flow-through aquarium system until the last week of their recovery period. They were then killed and dissected, and their shells were moved to a calcein bath (green rectangle) for one week until imaging. (3) In the stained live (SL) regime, mussels lived in the flow-through system until the last week of their recovery period, which they completed, live, in the calcein bath. They were then killed and imaged.

Table S1. Anova results comparing size distribution of mussels between treatments (stressed vs. non-stressed) and recovery durations (0, 1, 2, or 4 weeks) based on initial length and initial mussel wet weight.

	Morphological feature	Source	df	SS	MS	*F*	*P*
Initial sample	length (mm)	treatment	1	1	1.2	0.05	0.82
		week	3	15	5.1	0.23	0.87
		residuals	531	11514	21.7		
	wet weight (g)	treatment	1	1.3	1.3	0.58	0.45
		week	3	2.7	0.9	0.40	0.76
		residuals	531	1208.2	2.3		
After fatigue treatment	length (mm)	treatment	1	79	78.9	4.58	<0.05
		week	3	77	25.7	1.49	0.22
		residuals	229	3941	17.21		
	wet weight (g)	treatment	1	4.6	4.6	2.6	0.11
		week	3	9.1	3.0	1.7	0.17
		residuals	229	406.5	1.8		

Tests were conducted for groups as initially assigned (“initial sample”) and the final groups (“after fatigue treatment”), from which the shells that broke were excluded from the stressed group and the corresponding weakest shells were excluded from the control group.
